# Wake up and smell the dopamine: new mechanisms mediating dopamine activity fluctuations related to sleep and psychostimulant sensitivity

**DOI:** 10.1038/s41386-020-00903-5

**Published:** 2020-11-06

**Authors:** Andrew J. Kesner, David M. Lovinger

**Affiliations:** 1 Laboratory for Integrative Neuroscience, Division of Intramural Clinical and Biological Research, National Institute on Alcohol Abuse and Alcoholism, Bethesda, MD 20892 USA; 2 Center on Compulsive Behaviors, National Institutes of Health, Bethesda, MD 20892 USA

**Keywords:** Neuroscience, Medical research

Sleep serves a crucial survival function for species across the animal kingdom. Proper sleep not only provides time for rest and metabolic recovery via glial and endocrine functioning, but also contributes critical cognitive processes like memory consolidation and mood stabilization [1]. Indeed, as highlighted in a recent *Neuropsychopharmacology Reviews Series*, there is a growing consensus that most, if not all, neuropsychiatric disorders are associated with atypical sleep [2]. Also at the center of many of these psychiatric illnesses is the neuromodulator dopamine, which, in the central nervous system (CNS), has critical roles in movement control, reward and reinforcement, and affective processes [3]. Recent studies have also implicated dopaminergic activity in sleep [4] and have established that dopamine levels and release in the ventral striatum fluctuate in a circadian fashion [5, 6]. In rodents, sleep occurs in bouts that are most prominent in the light part of the cycle, but also occur in the dark period [1], and thus it was not clear from these earlier studies if dopamine fluctuations were related to sleep, time of day, or both. Furthermore, sleep in rodents can be separated into rapid-eye-movement (REM) and non-rapid-eye-movement (NREM) phases that can be distinguished from one another and from the awake state using polysomnography [1]. The dopamine transporter (DAT), which is responsible for removal/recovery of extracellular dopamine after release also exhibits circadian fluctuations in expression and function [5]. However, the mechanisms underlying these diurnal changes in dopamine and DAT activity and their relationship to sleep remain unknown.

In this issue of *Neuropsychopharmacology*, Alonso and colleagues [7] address these questions by measuring dopamine release at fixed time points after periods of sleep and wake states using brain slice fast-scan cyclic voltammetry (FSCV) in the core subregion of the nucleus accumbens (NAc core). This ventral striatal limbic CNS region was examined as it has prominent roles in control of affect and reward-driven behavior, and previous studies showed circadian fluctuations in dopamine in NAc core [5]. The authors also used immunological techniques to measure cell surface DAT expression and phosphorylation of the DAT protein at threonine 53 (pDAT), a posttranslational modification that enhances DAT function [8].

FSCV recordings indicated that the peak level of dopamine release induced by afferent stimulation (DA_STIM_) occurred shortly after a sleep bout during the light phase, but not the dark phase. Furthermore, the peak DA_STIM_ release was positively correlated with the number of REM bouts across both circadian phases. However, these DA_STIM_ transients were shorter in duration in brain slices examined after sleep bouts, relative to those examined after wake bouts, regardless of circadian period. Indeed, the rate of dopamine uptake was positively correlated with the percent time spent in either REM or NREM sleep, and negatively correlated with the percent of wake time. These findings indicate that sleep generally results in increased NAc core dopamine release in the light phase. However, increased dopamine uptake leading to a shorter duration of extracellular increases in dopamine likely leads to a net decrease in dopamine activity during sleep across the entire light-dark cycle.

The authors next examined changes in pDAT in relation to sleep. Increased pDAT was detected in relation to sleep, a finding consistent with enhanced DAT function that may explain the faster decay of stimulus-evoked dopamine release observed in rats that were recently sleeping.

Finally, the authors determined if the sleep-related changes in DAT and dopamine reuptake would alter sensitivity to a psychostimulant drug that acts on DAT, by examining effects of cocaine on the duration of DA_STIM_. Indeed, increased potency and efficacy of cocaine was observed in relation to sleep relative to the wake state, a highly translatable finding indicating that changes in DAT likely contribute to the sleep-related reduction in duration of DA_STIM_ increases.

The finding of enhanced dopamine uptake associated with sleep regardless of circadian phase provides valuable information about how this key neuromodulator may contribute to changes in NAc function in relation to disrupted sleep. Interestingly, the most consistent changes in dopamine were associated with REM sleep, including both increased release and reuptake. It will be of interest to determine the relevance of REM-related dopamine changes for behavior in future studies. For example, REM sleep is known to be profoundly altered in a variety of psychiatric illnesses, including depression and drug addiction [2]. Similarly, the amount of time spent in REM sleep is known to change throughout development and aging, indicating a potential understudied link to age-related psychiatric disorders. Indeed, the observation that cocaine’s effects are more pronounced during sleep may indicate that dopamine plays a role in susceptibility to drug actions and drug use in a manner that changes with altered sleep profiles. Thus, the relationship between time spent in different sleep phases and psychostimulant use will also be an important subject for future research.

It will be important to determine how the sleep-related changes in dopamine release and reuptake relate to in vivo levels of this key neuromodulator. One might predict lower levels of dopamine during NREM sleep relative to wake, perhaps associated with sharp, short-duration dopamine increases during REM sleep. In light of recent advances in the ability to measure dopamine changes in vivo using electrochemical techniques and genetically-encoded dopamine sensors [9] these studies are now feasible. It will also be interesting to determine how dopamine activity relates to experiences, such as stress and chronic intake of drugs of abuse, that are known to contribute to both sleep disruption and psychiatric illness, and the extent to which dopamine mediates their comorbidity. The association between sleep and pDAT levels indicates molecular mechanisms that may be targeted for altering sleep. It will be of interest to determine how perturbing DAT function alters sleep-wake balance as well as the duration and patterns of different sleep stages. The findings in the study by Alonso and coworkers thus set the stage for a variety of exciting future studies.

## Funding and disclosure

This work was supported by the Division of Intramural Clinical and Biological Research of the National Institute on Alcohol Abuse and Alcoholism (ZIA AA000416, DML PI) and a fellowship from the National Institutes of Health Center on Compulsive Behaviors (AJK).
